# Analyzing SARS-CoV-2 case numbers and clustering to predict a nursing home outbreak

**DOI:** 10.1007/s40520-025-03155-9

**Published:** 2025-08-31

**Authors:** Yasin Abul, Kevin McConeghy, Frank DeVone, Christopher Halladay, Stefan Gravenstein, James Rudolph

**Affiliations:** 1 Center of Innovation in Long Term Services and Supports, Providence VA Medical Center, Providence, RI USA; 2 Division of Geriatric and Palliative Medicine, Warren Alpert Medical School of Brown University, Providence, RI USA; 3 375 Wampanoag Trail, Suite 102, Riverside, RI 02915 USA

**Keywords:** SARS-CoV-2, COVID-19, Outbreak definition, Nursing homes, Older adults

## Abstract

The COVID-19 pandemic devastated nursing homes, highlighting the urgent need for effective outbreak control measures. This study analyzed twice-weekly PCR surveillance data from 134 Veteran Affairs Community Living Centers (December 2021-June 2022) to identify early predictors of SARS-CoV-2 outbreaks. Among 16,353 residents (mean age 74, 96% male, 68% white), we identified 1,868 infections and evaluated neighborhood ward-level case counts and their association with subsequent infections over two-week periods. Epidemic unit-days with no initial cases had an 87.49% likelihood of remaining case-free, while those with ≥ 4 initial cases demonstrated a 38.5% probability of developing ≥ 4 additional cases. These findings indicate that early case clusters strongly predict larger outbreaks, underscoring the importance of rapid detection and intervention. Study limitations include demographic homogeneity and reliance on frequent PCR testing, potentially limiting generalizability. This research provides a valuable framework for refining outbreak definitions and improving infection control strategies for respiratory virus outbreaks in nursing homes.

## Introduction

Severe Acute Respiratory Syndrome Coronavirus 2 (SARS-CoV-2) emergence devastated U.S. nursing homes (NHs), killing over 170,000 NH residents [1]. Early mortality mounted despite clinical triaging and isolation of ill residents for outbreak control, leading healthcare provider and policymakers to recognize that:

a) symptom-only screening failed to identify half of residents with SARS-CoV-2 early in the pandemic [2]; b) ≥ 40% of healthy adults were asymptomatic [3]; and, c) asymptomatic and presymptomatic individuals could transmit virus and spread infection [4]. It was not until outbreak management had stabilized and vaccines became available that we could systematically examine the timing of infection control measures in nursing homes.

To systematically examine the optimal timing for triggering infection control practices in nursing homes, several key elements of outbreak response must be aligned across study sites. This includes harmonizing both the data and operational practices. Specifically, we focused on VA CLC facilities using: (a) routine, facility-wide PCR-based SARS-CoV-2 surveillance testing of all residents—rather than limiting testing to those with symptoms or using only rapid antigen tests; (b) a unified electronic health record system to track and analyze infections over time; and (c) VA nursing homes with similar operational structures, including similar policies on staffing and infection control.

To inform timely interventions, infection control measures should be based on an early signal that reliably predicts an impending outbreak. However, defining the threshold for such an outbreak has varied widely and often depends on local viral transmission, variant types, speed of case identification, and vaccination coverage [4]. Using data from Veterans Affairs (VA) Community Living Centers (CLCs) as NH analogues, we evaluated how the number and timing of initial SARS-CoV-2 cases could be used to predict the likelihood of additional cases within a facility.

## Methods

We utilize data collected in the Veteran Health Administration’s 134 CLCs for routine resident care. The Providence Institutional Review Board determined this minimal risk study exempt from review. We captured all twice-weekly SARS-CoV-2 PCR surveillance and symptomatic test results on residents by CLC neighborhood (specialized ward of a grouping in one of three CLC households). We define SARS-CoV-2 activity as the sum of PCR-confirmed SARS-CoV-2 infections (PCR+) in the prior 7 days of an index date within a so-called epidemic unit, a predefined geographic area–neighborhood-ward– used to track disease spread (epidemic unit = neighborhood-ward). Our unit of observation is the epidemic unit-day, that is a single day from an eligible epidemic unit; from there we look back 7 days and count the number of positive SARS-CoV-2 PCR tests that were present (inclusive of the day of, so 8 days total). We then look forward two weeks and count the number of subsequent positive SARS-CoV-2 PCR tests that appear. The choice of numbers for initial/additional cases as the cut off point, as well as the 7-day and 2-week intervals, was data-driven and based on observed patterns within the study population. Specifically, the 7-day look-back window aligns with the typical incubation period for SARS-CoV-2 and is consistent with surveillance intervals in nursing home settings [5]. The 2-week look-forward period reflects the standard monitoring window for outbreak progression and aligns with public health guidelines for outbreak management [6]. This is a single observation for our analysis and is repeated for each day and each epidemic unit from 12/18/2021 to 06/18/2022. The methodology assumes that true outbreaks will manifest as locally and temporally concentrated case clusters, whereas sporadic cases from prior epidemics would lack both the spatial clustering (same ward) and tight temporal sequence (7-day window) required to meet the outbreak definition. Routine standard of care outbreak control measures implemented in VA Community Living Centers during the study included resident and staff symptom screening, use of personal protective equipment, isolation and cohorting of cases, suspension of group activities and communal dining, and restrictions on visitation in accordance with established long-term care protocols.

## Results

**Fig. 1:**
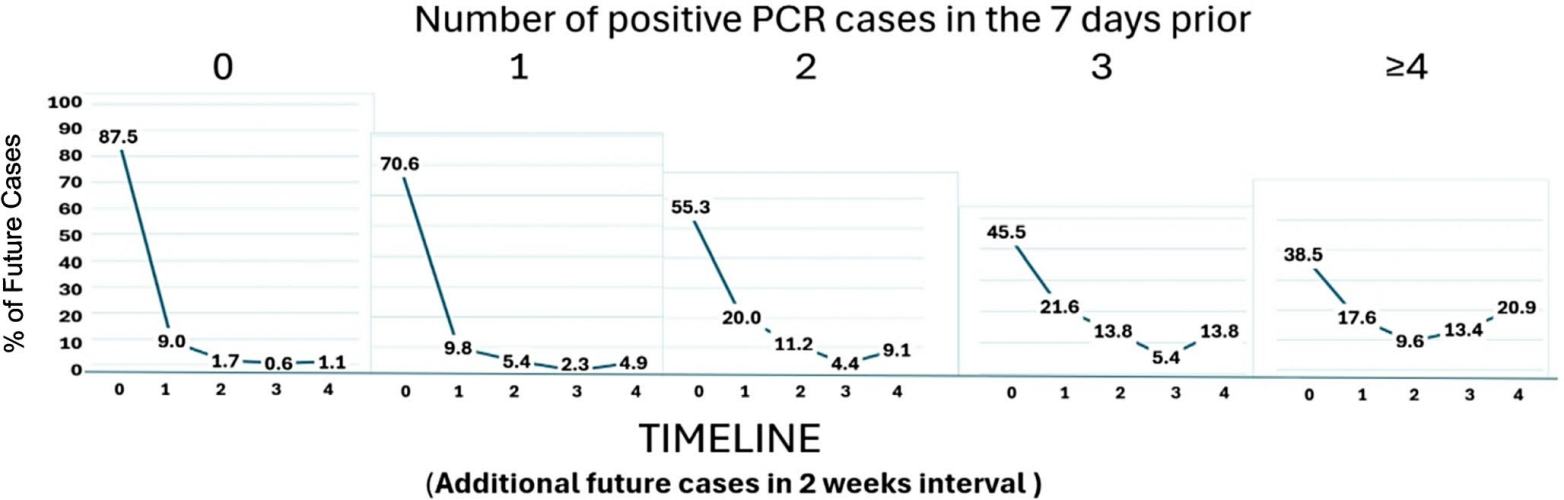
Percentage of additional SARS-CoV-2 cases within two weeks based on the number of positive PCR cases in the prior seven days* * For each ward(epidemic unit) on a specific day, we count the total number of positive SARS-CoV-2 PCR tests reported over the past 7 days, including that day (an 8-day period in total). Then we look forward 2 weeks to count how many new positive tests occur in that same ward(epidemic unit). This process creates one observation for our analysis. By repeating this for every epidemic unit and every day between Dec, 19, 2021 and June, 18, 2022, we aim to understand how to number of recent cases might relate to future casein those wards(epidemic units)

The 134 CLCs housed 16,353 Veterans, mean age 74, mostly male (96%) and white (68%). We evaluated 490 epidemic units with an average daily census of ≥ 3 from 18DEC2021-18JUN2022. We identified 1,868 SARS-CoV-2 infections amongst 1,769 unique Veterans. The primary outcome was the PCR + cases per epidemic unit day in a two-week period. Absent initial PCR + cases in a given epidemic unit-day 87.49% of epidemic unit-days had no additional PCR + in the next two weeks, while the incidence of 4 or more additional PCR + individuals was only 1.10%. As the initial PCR + individual count increased, so did the likelihood of more cases in the subsequent two weeks. For example, with ≥ 4 initial positives, 38.5% of instances had ≥ 4 additional positives in the next two weeks in an epidemic unit. This threshold was empirically determined to best distinguish between smaller clusters and those more likely to escalate into larger outbreaks. The mean number of new cases observed in two weeks went from 0.21 (when the epidemic unit-day has no observed PCR+) to 2.0 (when the epidemic unit-day has 4 or more observed PCR+) (Fig. 1). Vaccine uptake percentages were presented in Table 1.

**Table 1:** COVID-19 vaccination status among VA community living center (CLC) residents during study period

Study period	Total residents	Fully vaccinated residents	Residents received at least one booster	Residents received at least two boosters
December 2021-June 2022	16,353	14,050 (86%)	11,509 (70%)	5303 (32%)

**Fully vaccinated**: Residents who completed a primary COVID-19 vaccine series (2 doses of mRNA vaccine) with at least 14 days having passed since the final dose

**Up to date for vaccination**: Residents who were fully vaccinated and received at least one booster dose (per CDC guidance during that period)

## Discussion

The study demonstrated an association between initial SARS-CoV-2 case counts and the probability of further transmission, indicating a predictive case-cluster for outbreaks. These findings suggest that early detection of positive cases can serve as a signal for implementing enhanced outbreak control measures in nursing homes.While the approach provides a framework, we acknowledge that outbreak dynamics are also influenced by factors such as the basic reproduction number (R0), testing frequency, and pathogen-specific characteristics.The study’s framework for predicting SARS-CoV-2 outbreaks in nursing homes can be adapted to other pathogens by integrating detection techniques (i.e. on site point of care testing), incorporating pathogen-specific transmission dynamics(i.e. virus variants) and setting specific calibrations (i.e.nursing home specific factors).

### Strengths and limitations

The large geographic spread of the VA CLC system provides a nationwide sample, which is a strength of this study. The study population was predominantly white, non-Hispanic male veterans living in CLCs with unique staffing patterns and physical layouts. These differences from community nursing homes may limit how well the findings apply to other demographic groups. The reliance on twice-weekly PCR testing may mean that fewer cases could serve as a tipping point for outbreaks if less aggressive surveillance is used. This could influence the sensitivity of outbreak detection in settings with different testing frequencies such as in community NHs.

Data collection over a six-month period might not capture seasonal variations in SARS-CoV-2 transmission, potentially affecting the study’s applicability across different times of the year. Lastly, variations in cognitive functions among patients significantly affects the effectiveness of control measures.

## Data Availability

The secondary analysis of clinical data was approved by the Providence VAMC IRB and R&D committees. Data release is governed by the VA Data Policy and is unable to be released at this time.

## Declarations

This work was submitted to be published as an abstract to IDWeek.
